# Surveillance of the recurrence time of the effectiveness of national and region-level quarantines of COVID-19 pandemic in Chile

**DOI:** 10.1371/journal.pone.0295368

**Published:** 2024-03-20

**Authors:** Bernardo Lagos-Álvarez, Salomé Zaldúa Flores, Jorge Figueroa-Zuñiga, Francisco Novoa-Muñoz

**Affiliations:** 1 Departamento de Estadística, Facultad de Ciencias Físicas y Matemáticas, Universidad de Concepción, Concepción, Chile; 2 Departamento de Enfermería, Facultad de Ciencias de la Salud y de los Alimentos, FACSA, Universidad del Biobío, Campus Fernando May, Chillán, Chile; Universidad Santo Tomas, CHILE

## Abstract

The World Health Organization has recommended a range of social and health measures to mitigate the spread of coronavirus disease 2019 (COVID-19), including strategies such as quarantines, border closures, social distancing, and mask usage, among others. Specifically, the Chilean authorities implemented the “step-by-step” plan, built on the concept of dynamic quarantine. Numerous studies have examined the effectiveness of these quarantines in Chile during the pandemic, utilizing data published by the Chilean Ministry of Health. This study’s primary aim was to enhance our understanding of quarantine effectiveness in Chile during the pandemic. We accomplished this by analyzing the distributional behavior of the time until the COVID-19 pandemic was deemed under control or not. In our study, we defined an event with two potential outcomes related to the instantaneous reproductive number (*R_t_*), which signifies the time until a change in the event outcome occurs. Importantly, we did not predefine a specific temporal observation unit. These findings allowed us to complement the concept of effective quarantine by considering the dynamics generated by the protocols, such as phase 1 of the quarantine, in achieving natural herd immunity in response to the number of COVID-19 cases and *R_t_*. We assessed the behavior of the mean and median residual lifetime until the initiation of controlled/uncontrolled episodes of the COVID-19 pandemic based on *R_t_* in all regions of Chile. Despite variations in the distribution of residual times for controlled/uncontrolled episodes in different regions, there was a similar observation during the period considered (between March 2020 and March 2021): the mitigation measures did not produce a clear positive effect for controlling the epidemic. The residual times of episodes with *R_r,t_* > 1 were not different from those episodes with *R_r,t_* ≤ 1.

## Introduction

Since the initial reports of COVID-19 cases emerged from Wuhan, China, in December 2019, the global impact of this disease has been profound [1, 2]. As of the latest data, there have been more than 757 million confirmed cases of COVID-19 and approximately over 6.8 million deaths worldwide, with the Americas accounting for 43% of the total fatalities [3].

To combat the spread of COVID-19 and minimize the risk of severe illness, the World Health Organization (WHO) has recommended a range of preventive measures, such as physical distancing, quarantines, border closures, mask usage, frequent hand washing, and vaccination, among others.

Chile is divided into 16 political-administrative regions, each comprising multiple communes administrated by municipalities. Throughout the pandemic, individual communes in Chile adopted varying quarantine protocols tailored to the specific circumstances and conditions in their respective areas. The Chilean authorities introduced a “step-by-step” plan based on the concept of dynamic quarantines. Comprehensive details can be found here: https://www.gob.cl/pasoapaso.

This plan initially consisted of five phases, with mobility restrictions imposed at the municipal level based on infection rates in each region. These measures were initiated towards the end of July 2020. The final phase, Phase 5, known as “advanced opening”, signified the end of quarantine measures for the entire population and was implemented nationwide from October 1, 2022.

Our study focused on data collected during the observation period spanning from March 3, 2020, to March 3, 2021, providing a one-year dataset. Notably, on December 24, 2020, a nursing assistant became the first person to receive the COVID-19 vaccine in Chile. Mass inoculation commenced on February 3, 2021, and, within 21 days, approximately 16% of the population had been vaccinated. Chile was among the countries that achieved herd immunity relatively early and efficiently [4].

For the purpose of our analysis, we specifically concentrated on the initial two phases of the “step-by-step” plan, which we will describe in detail:

- Phase 1 (Quarantine). Implies total and permanent confinement, with restricted mobility and special permits for essential activities. There is a permanent and mandatory quarantine for adults over 75 years old. Social and recreational activities and the operation of restaurants, cinemas, and theaters are prohibited to minimize the interaction and spread of the virus.
- Phase 2 (Transition). Decreases the degree of confinement. There is a permanent and mandatory quarantine for adults over 75 years old. Movement is allowed from Monday to Friday for the population under 75 years of age, and social and recreational activities can be carried out during the week, with a maximum number of 10 people. The operation of restaurants, cinemas, and theaters is prohibited to avoid sudden mobility and minimize the risks of contagion.

Studying and comprehending the efficacy of protocols implemented to manage infections during an epidemic, along with their consequences for the population, is of paramount importance. Long-term protocols, particularly total confinement measures, represent extreme actions that carry psychological and economic burdens for both the populace and various productive sectors [5].

Grigorieva et al. (2020) [6], in their research on the effectiveness of quarantine over time using the SEIR method, modeled the performance of quarantines with varying durations, considering different approaches. Among their key findings, they demonstrated that quarantine measures are highly effective during the initial month of implementation, with an effectiveness rate of 0.9. However, as quarantines extend beyond this point, their effectiveness diminishes, reaching levels as low as 0.2 by the conclusion of the second month. Sjödin et al. (2020) [7], also employing the SEIR method, introduced a new sociodemographic variable to evaluate the effectiveness of quarantine measures. Their research highlighted the necessity for substantial community adherence to quarantine measures to control the rise in infections. Moreover, they identified that the size of households in areas where these measures are enacted plays a pivotal role. Their conclusion was that larger average household sizes necessitate more extended quarantine periods to effectively manage the spread of the virus. Given that quarantine measures are inherently social, it is reasonable to assume that their effectiveness is contingent upon various factors, including the level of public acceptance, shifts in adherence over time, and sociodemographic characteristics, such as average population density and overcrowded living conditions.

Numerous studies have scrutinized the efficacy of quarantine measures implemented in Chile during the pandemic, drawing from data published by the Chilean Ministry of Health [8–11]. These investigations have embraced diverse methodologies.

For instance, Kristjanpoller et al. (2021) [10] introduced a comprehensive framework aimed at evaluating the effectiveness of the dynamic quarantine policy. Their approach involved an analysis of various causal models dependent on 17 covariables, with the quarantine state (status) serving as the treatment variable. To achieve this, they harnessed a range of machine learning models, capable of conducting causal inference when the requisite conditions are met. This encompassed an examination of time series patterns linked to the effective reproductive number. The analysis was primarily centered on the municipal level in Chile, identifying that the most effective base model was the XGBoost in the X-learner framework. Nevertheless, it’s worth noting that the study did not address the modeling of the duration of states with *R_t_* > 1 or *R_t_* ≤ 1 in conjunction with the periods during which quarantines were enforced. Grebe et al. (2020) [11] furnished a comprehensive overview of the impact of nationwide measures implemented during the initial months of the pandemic. They assessed these measures using the daily transmission rate, with a particular emphasis on the Metropolitana region, where is Santiago, the capital city of Chile, and approximately 42% of Chile’s population. Various mathematical models were fine-tuned to elucidate and forecast the course of epidemics. Some of these models incorporate daily infection reports, including confirmed cases (positive PCR tests) from day one and the number of historical cases, among other variables. These models often calculate epidemiological indicators that guide mitigation measures, such as the reproduction number, which characterizes the epidemic’s transmission potential.

There are two types of reproduction numbers: the Basic Reproduction Number (*R_0_*) and Instantaneous (or effective) Reproduction Number (*R_t_*). *R_0_* signifies the average number of secondary infection cases that arises in a completely susceptible population ollowing the introduction of a single infectious case. On the other hand, *R_t_* represents the average number of infection transmissions originating from each infectious case, taking into account the current level of immunity in the population within a specific time frame [12, 13].

To gauge the effectiveness of public health interventions, it is imperative to quantitatively assess *R_t_* across variou settings, ideally at regular and frequent intervals (daily or weekly) [14].

In our study, we introduce an event with two possible outcomes linked to *R_t_*, capturing the duration until a change in the event’s status occurs, all without imposing any predefined temporal observation unit. These findings, we believe, provide a unique perspective that enriches the concept of effective quarantine. By considering the behavioral dynamics influenced by the protocols, including phase 1 of the quarantine, our study aims to contribute to the understanding of how natural herd immunity may be attained in response to the number of COVID-19 cases and *R_t_*. This novel approach underscores the core innovation of our research.

Hence, the principal objective of our study centers on advancing our comprehension of quarantine effectiveness in Chile during the pandemic. We achieve this by quantitatively evaluating the time remaining until the end of the study period, taking into account both states of epidemic control and non-control, across various regions.

The time until a specific event occurs is a variable encountered in various analyses. Survival analysis is a statistical method for analyzing failure time data, providing straightforward and intuitive results regarding time-to-event for events beyond just “death”. Residual lifetime analysis has been used in various fields, including engineering, survival analysis, economics, and actuarial science, for many years. The mean residual lifetime (*MRL*) represents the average time until an event occurs, given that an individual has survived up to a certain time. For an in-depth discussion of the statistical applications of the *MRL*, please refer to Embrechts et al. (1997) [15]. The median residual lifetime (*MERL*) can be estimated using a survival curve and identifies the time point at which the survival probability is 0.5. *MERL* is often preferred over MRL because it is less influenced by extreme values of the variable, as explained in Kidwell et al. (2014) [16] and Schmittlein and Morrison (1981) [17]. Both *MRL* and *MERL* are frequently used as summary measures to assess treatment effectiveness. However, there is limited literature on the combination of reproduction number series and residual time analysis, making our study a valuable contribution in this direction.

We achieve our objective by incorporating the instantaneous reproductive number, which considers the speed at which a disease spreads in a population. We use *MRL* and *MERL* to evaluate whether the mitigation protocols have effectively controlled the pandemic. Effective quarantine aims to limit the unrestricted spread of the disease, thereby keeping the instantaneous reproductive number below one to achieve controlled spread, as described [18]. This approach to surveillance of pandemic mitigation effects has not been extensively employed in evaluating quarantines during the COVID-19 pandemic. We believe that our study justifies the need to develop methods that realistically consider the residual times of the event of interest, without reaching an exhaustive predictive objective, where the phennomen becomes repetitive from the inception of the datas registration. Furthermore, this phenomenon is subject to continuous intervention.

The structure of this paper is organized as follows: Section 2 provides a concise description of the data used in this study, with a focus on the instantaneous reproductive number index generated in studies by [9]. We also explain the methods related to estimating the mean and median of the residual time until the control or non-control of the pandemic occurs. Section 3 presents the results, while Sections 4 and 5 delve into the discussion and conclusion of this study.

## Materials and methods

### Variables considered and data description

To assess the effectiveness of the quarantine measures imposed in Chile with regard to daily infection numbers in each region, we employed a descriptive analysis and a time-residual analysis commonly utilized in survival analysis.

**Fig 1:**
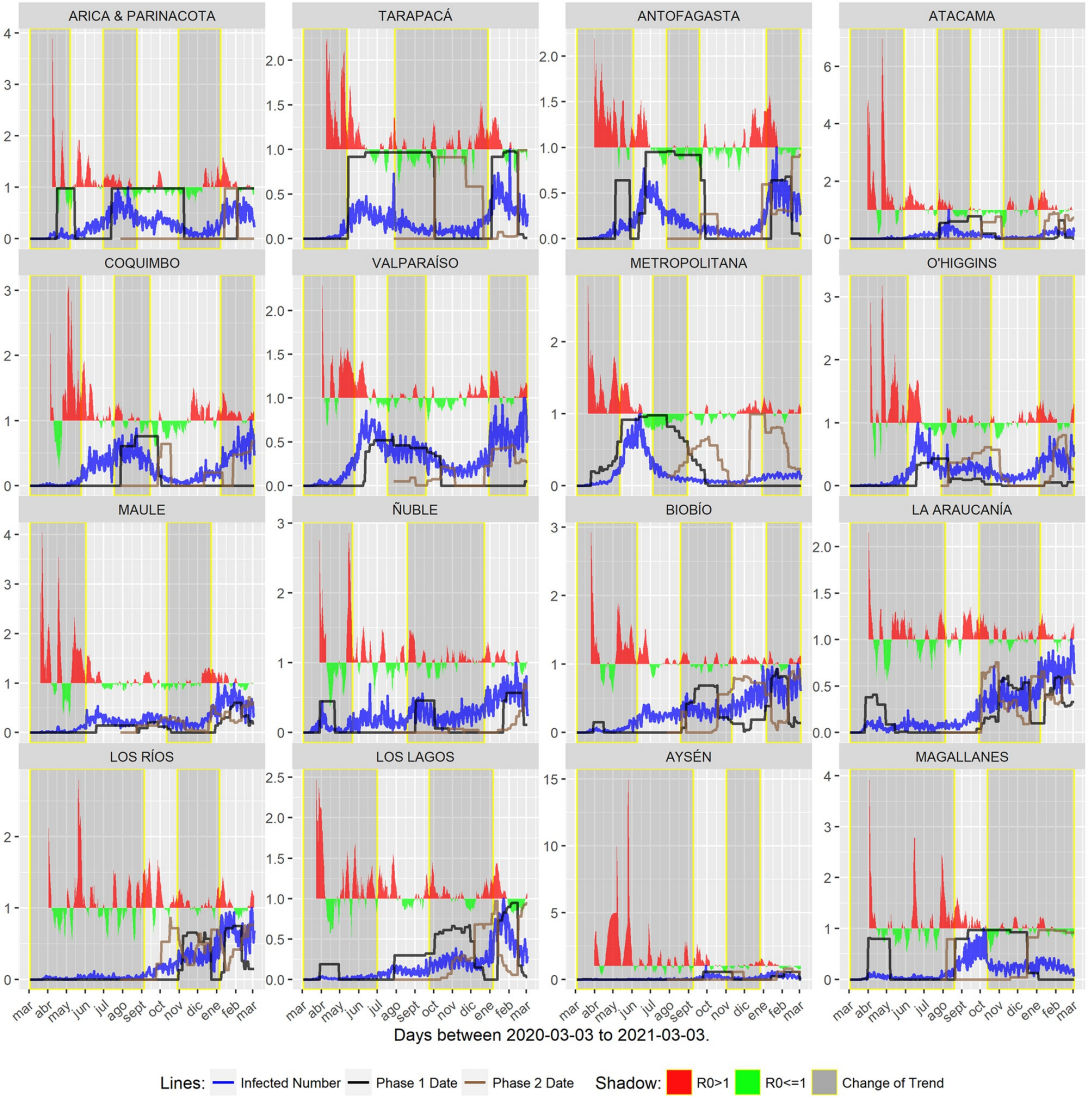
Time series of *R*_*r*,*t*_, *Y*_*r*,*t*_ and $X_{r,t}^{\left(j\right)}$. The daily percentage of COVID-19 confirmed cases, broken down by region, denoted by the blue line. The black and brown lines depict the percentage of people under Phases 1 and 2, respectively. The red curve represents the instantaneous reproduction number *R_t_* >1, while the green curve indicates *R_t_* ≤ 1. The yellow-shaded grey bars highlight periods in the time series when there was a shift in the trend of confirmed COVID-19 cases. Source: Authors.

This study utilized daily COVID-19 pandemic records in Chile, alongside specific variables managed by the Chilean Ministry of Science, Technology, Knowledge, and Innovation. These variables included the percentage of COVID-19 confirmed cases and the percentage of the population in regions of Chile under Phases 1 and 2 restrictions. The data spanned from March 2020 to March 2021. The data were extracted from the COVID-19 Data Table, accessible at https://www.gob.cl/pasoapaso/cifrasoficiales/ (accessed on 22 April 2021). These datasets were analyzed in previous research conducted by Barría-Sandoval et al. (2022) [8] and Jerez-Lillo et al. (2022) [9]. A summary of the information extracted from these studies is presented in Fig 1.

The daily counts of COVID-19 confirmed cases, disaggregated by region from Arica and Parinacota to Magallanes, are denoted as ${\tilde{Y}}_{r,t}$ where *r* = 1, …, 16 and *t* ∈ {2020- 03- 03, …, 2021- 03- 03}. These counts were transformed into the series *Y*_*r*,*t*_ while maintaining the same values for *r* and *t* and indicated by the blue line in Fig 1. The transformation was executed as follows: $Y_{r,t}=\frac{{\tilde{Y}}_{r,t}-a}{b-a}$, whith *a* representing the minimum value of $\text{min}\left\{{\tilde{Y}}_{r,t}\right\}$ and *b* representing the maximum value, ensuring data consistency and comparability. The black and brown lines in in Fig 1 represent the percentage of people in Phases 1 and 2, respectively, corresponding to the time periods when the protocol interventions were in effect. These time series, denoted as $X_{r,t}^{\left(j\right)}$, *r* = 1, …, 16, *j* = 1, 2, *t* ∈ {2020-03-03, …, 2021-03-03}, are calculated as follows:

$$\begin{array}{c} X_{r,t}^{\left(j\right)}=\sum_{c\in r}\frac{P_{c}}{\sum_{c\in r}P_{c}}1\left(F_{c,t}=j\right),\;j=1,2, \end{array}$$ (1)

In this expression, *P_c_* represents the population of commune *c*, ∑_*c*∈*r*_ *P*_*c*_ is the total population of region *r*, *F_c,t_* signifies the phase of the step-by-step plan for commune “*c*” at time *t* and 1(·) serves as an indicator function, defined as:

$$\begin{array}{c} 1\left(F_{c,t}=j\right)≔\{\begin{array}{cc} 1\; & \;\text{if}\;\;F_{c,t}=j,\\ 0\; & \;\text{if}\;\;F_{c,t}\neq j. \end{array} \end{array}$$ (2)

This formula allows us to compute the percentage of people in Phases 1 and 2 based on the population distribution and the phase of the step-by-step plan for each commune and region.

Furthermore, Fig 1 displays the breakpoints of the COVID-19 confirmed cases trend using gray bars in each region’s chart. These bars represent the periods during which changes in the trend of confirmed COVID-19 cases occurred. Additional information regarding the identification of these periods can be found in in Jerez-Lillo et al. (2022) [9].

The instantaneous reproduction number, *R_t_*, is estimated using a Bayesian approach, with its posterior median represented in Fig 1. Details of this estimation process are provided in Jerez-Lillo et al. (2022) [9] and Cori et al. (2013) [19]. In Fig 1 *R_t_* ≤ 1, where *r* = 1, …, 16 and *t* ∈ {2020-03-03, …, 2021-03-03} is depicted by a green curve, indicating that the measures implemented have been effective in controlling the pandemic. Conversely, the red curve represents *R_t_* > 1, with the same values for *r* and *t*, suggesting that the measures have not been effective in controlling the pandemic.

It’s important to note that providing a detailed description of the different phases (phases 1 to 5) linked to the calendar year for each region is challenging due to variations in protocol implementation dates across municipalities within each region.

All the analysis was developed in the R software (R Core Team and the R Foundation for Statistical Computing, Vienna, Austria), which is available at https://www.R-project.org/.

### The residual lifetime

Suppose we have a continuous non-negative random variable *T* with a cumulative distribution function *F_T_*. The residual lifetime random variable at a specific time *t*_0_ is denoted as $T_{t_{0}}$, defined as *T* − *t*_0_|*T* > *t*_0_. This represents the additional lifetime of an item, given that it has survived up to time *t*_0_.

As *R_t_*, it signifies the expected number of secondary cases originating from an initial case at time *t*. This estimate varies over time during the outbreak. When *R_t_* ≤ 1, the outbreak is likely to be controlled, however, if *R_t_* exceeds 1, it indicates a sustained infection rate. The primary goal of control measures is to reduce *R_t_* to less or equal to one.

Now, we can define two variables for each region: representing the time until the spread of the virus is controlled (*R_t_* ≤ 1) and uncontrolled (*R_t_* > 1). For simplicity, let’s call these variables “OK”/”NOK”, respectively. In terms of data:

-
$T_{r,l_{r,t}}^{OK}$ represents the number of consecutive days with *R*_*r*,*t*_ ≤ 1, *l*_*r*,*t*_ = 1, …, *n*_*r*,*N*_
-
$T_{r,j_{r,t}}^{NOK}$ represents the number of consecutive days with *R*_*r*,*t*_ > 1, *j*_*r*,*t*_ = 1, …, *m*_*r*,*N*_

where *l*_*r*,*t*_ and *j*_*r*,*t*_ are the indices represent the periods when there are consecutive days with *R*_*r*,*t*_ ≤ 1 and *R*_*r*,*t*_ > 1, respectively. Here, *n_T_* and *m_T_* are the observed quantities of $T_{r,l_{r,t}}^{OK}$ and $T_{r,j_{r,t}}^{NOK}$, respectively, for different values of *R_t_*, denoted as *N*.

The mean residual lifetime at time *t*, denoted by *MRL(t)*, measures the expected remaining lifetime of an event of interest for an individual day *t*. If *E*_*F*_{*T*} < ∞ it is defined as:

$$\begin{array}{c} MRL\left(t\right)=\{\begin{array}{cc} E_{F}\left\{T-t|T>t\right\}, & \text{for}\;\text{those}\;t\;\text{such}\;\text{that}\;S(t)>0,\\ 0, & \text{for}\;\text{those}\;t\;\text{such}\;\text{that}\;F(t)=0, \end{array} \end{array}$$ (3)

In mathematical terms, where *T* represents a lifetime (i.e., a nonnegative random variable) and *S(t)* is the survival function of *F_T_* at time *t*, we can interpret this as the expected additional time during which the instantaneous reproduction number will remain greater than 1, considering that the time when the instantaneous reproduction number was greater than 1 extends beyond *t*.

The median residual life (*MERL*) is given by

$$\begin{array}{c} MERL(t)=\text{median}\{T-t|T>t\}, \end{array}$$ (4)

which can be expressed as *MERL*(*t*) = *S*^−1^(*S*(*t*)/2) − *t*. provides insight into the median additional time during which the instantaneous reproduction number remains greater than 1, considering that the time when the instantaneous reproduction number was greater than 1 extends beyond *t*. We can also write *x* ≔ *MERL*(*t*) as the solution to 1 − *F*(*t* + *x*) = 0.5 × (1 − *F*(*t*)) (see [20]).

To illustrate the differences between the mean of *T* and the *MRL(t)* of time *T*, let’s consider an example using the density function, *f*(*t*) = 3(100*t* − 20*t*^2^ + *t*^3^)/2500, 0 < *t* < 10. In this case, we have *E*{*T*} = 4, *E*{*T*|*T* > 2} = 4.6 and *MRL*(2) = 2.6. Additionally, the median{*T*} ≈ 3.857276, median{*T*|*T* > *t*} ≈ 4.387201 and for *MERL*(2) ≈ 2.387202. The impact that these differences have will be assessed according to the area of study. However, in the area of health they become important.

For the non-parametric approximation of sample functions of *MRL* and *MERL* is obtained by substituting the Kaplan–Meier estimator of *S*(*t*), or of *F*(*t*) (see [21]). Numerically it can be achieved by using the MMRtime function of the emplik package of R (see Zhou and Yang [22]) that is based on the work of Zhou (2016) [23].

In the context of COVID-19, analysis of median and mean time residuals can be a valuable approach to assess the effectiveness of quarantine measures over time. This analysis involves examining the curves of median and mean time-residuals across different regions and their relationship with the proportions of citizens complying with quarantines. Focusing on Phase 1, this analysis aids in evaluating the model’s predictive reliability and provides valuable insights for decision-making regarding resource allocation, public health interventions, and policy measures. It also enables researchers to refine and enhance models for improved predictive accuracy in future scenarios.

## Results

### Analysis of sample *MRL* and *MERL*

**Fig 2:**
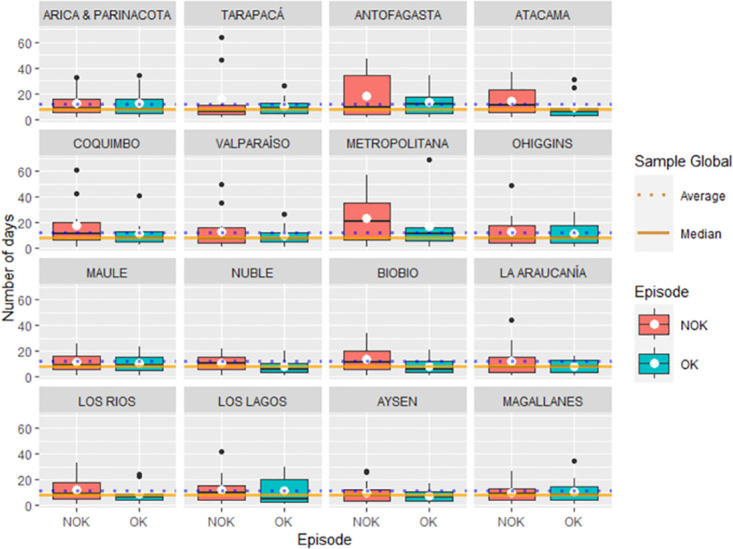
Box-plot of the number of consecutive days of *R*_*t*_ ≥ 1 and *R*_*t*_ > 1. Box-plot of ${\text{T}}_{r}^{OK}$ and ${\text{T}}_{r}^{NOK}$, *r* = 1, …, 16. Source: Authors.

With the generated datasets ${\text{T}}_{r}^{OK}≔\left\{T_{r,l_{r,t}}^{OK};l_{r,t}=1,\ldots ,n_{r,N}\right\}$ (despict green color) and ${\text{T}}_{r}^{NOK}≔\left\{T_{r,j_{r,t}}^{NOK};j_{r,t}=1,\ldots ,m_{r,N}\right\}$ (despict red color), shows over Fig 2, we can then perform a comparative analysis to the variables, the number of consecutive days with *R*_*r*,*t*_ ≤ 1 against the number of consecutive days with *R*_*r*,*t*_ > 1, utilising a box-plot (Fig 2). It’s evident that the behaviors of ${\text{T}}_{r}^{OK}$ and ${\text{T}}_{r}^{NOK}$ vary across regions. When comparing ${\text{T}}_{r}^{OK}$ and ${\text{T}}_{r}^{NOK}$ separately for *MRL* and *MERL* within each region, we found no significant differences (p-value > 0.05, based on classical t-test and Wilcoxon test) indicating that the number of consecutive days where *R_r,t_* ≤ 1 and *R_r,t_* > 1 are statistically similar in each region.

Descriptively, the box-plot suggests that the implementation of protocols in the Tarapacá, and Los Lagos regions was effective, as evidenced by a longer duration of *R_r,t_* ≤ 1 (“OK” event) was longer. Conversely, the mean duration of *R_r,t_* > 1 (“NOK” event) was longer in three regions: Antofagasta, Atacama, and Metropolitana regions.

**Fig 3:**
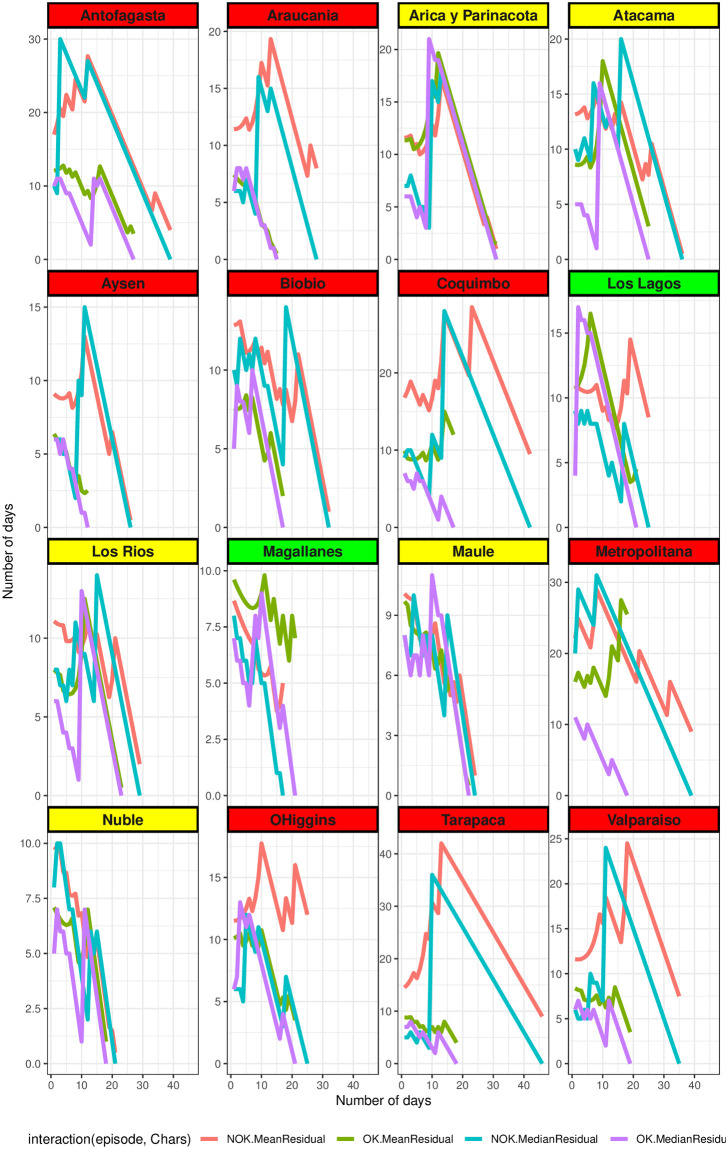
Estimate of the mean and median residual time according to “OK/NOK” events. Estimate curve of the mean (*MRL*) and median (*MERL*) residual time of the “OK/NOK” events for each region of Chile. Source: Authors.

Additionally, we present the estimated mean and median of the remaining duration of the events in Fig 3. In this figure, it is evident that the curves for the “OK” event ($T_{r,l_{r,t}}^{OK}$) represented by the olive green curve for *MRL* and the purple curve for *MERL*, are consistently above the other curves. This suggests a trend towards containing the pandemic for the remaining time periods.

Conversely, the estimated average duration of the “NOK” event ($T_{r,j_{r,t}}^{NOK}$) represented by the red curve for *MRL* and the turquoise curve for *MERL*, given that the duration is longer than *t* (*t* = 1, …, 33), is longer for nine regions; Antofagasta, Tarapacá, Coquimbo, Valparaiso, O’Higgins, Metropolitana, Biobio, Araucania and Aysén regions. Compared to the controlled pandemic episodes (“OK” event). These findings suggest that the estimated mean and median durations of uncontrolled pandemic episodes (“NOK” event) were longer than those of controlled episodes (“OK” event) achieved through the implementation of measures. This may imply that the disease showed resistance to the mitigation measures, or the measures applied were not robust enough.

In summary, when considering both Figs 2 and 3, it becomes clear that in most regions, the estimated mean and median durations of the “NOK” episode are greater than those obtained for the “OK” episode.

Fig 3 provides valuable insights into the expected behavior across most regions. It illustrates that as we extend the defined time parameter *t* for the event *T* > *t*, the corresponding estimates gradually decrease, ultimately approaching zero. Furthermore, the figure reveals noteworthy distinctions between the curves of *MRL* and *MERL*, highlighting the influence of outliers in various regions. Notably, for “OK” events, the regions of Coquimbo, Metropolitana, and Magallanes exhibit notable outliers, while for “NOK” events, the regions of Tarapacá, Coquimbo, O’Higgins, Araucanía, and Los Lagos display similar outlier patterns.

A closer examination of the Maule region from Fig 3 does not clearly indicate that the measures implemented are having a positive effect. Furthermore, Fig 1 shows that both the black (representing Phase 1 application) and brown (representing Phase 2 application) curves closely follow the behavior of the transformed number of positive cases (blue curve), which hinders pandemic containment. The duration of periods with *R_r,t_* ≤1 noteworthy. Similar trends are observed in the Arica, Parinacota, and Ñuble regions.

In contrast, for the Tarapacá region, the effect of Phase 1 protocol application is minimal (black curve), and it does not align with the behavior of the transformed number of positive cases (blue curve). This discrepancy arises because the *R_r,t_* curve remains above one for an extended period. A similar situation is observed in the Antofagasta region.

Upon closer examination, we can conclude that when the pandemic remains uncontrolled (“NOK”) for more than 15 days, the average estimated remaining time (until March 3, 2021) in the uncontrolled state is significant. For instance, in the Magallanes region, it is approximately 4.3 days, while in the Metropolitan region, it extends to 23 days. Conversely, when the pandemic remains controlled (“OK”) for more than 15 days, the average estimated remaining time in the controlled state is notable. In the Magallanes region, it’s approximately 8 days, and in the Metropolitan region, it’s 19 days.

Remarkably, the olive-colored curve consistently appears above the purple-colored curve in the Magallanes region. Conversely, in the Metropolitana region, the olive-colored curve mainly lies below the purple one. These statistics suggest that the duration of the “NOK” event is significantly longer in the Metropolitan region compared to the Magallanes region.

These findings indicate that implementing mitigation protocols in the Magallanes region, as compared to the Metropolitan region, results in a significant difference in the estimated average remaining time until March 3, 2021, for a controlled quarantine. Specifically, when the duration of the uncontrolled quarantine exceeds 15 days, the Magallanes region exhibits an average reduction of 18.5 days compared to the Metropolitan region. In other words, the mitigation protocols implemented produce a difference in the estimated average remaining time in the Magallanes region, reducing it by 11 days (= 8-19) compared to the Metropolitan region, when the duration of the controlled quarantine exceeds 15 days.

Notably, the percentage of people in each region participating in phases 1 and 2 is higher in the Magallanes region, and their participation it begins earlier. If we analyze the median curve in the Los Lagos region (purple curve, representing “OK” events), it consistently surpasses the green median curve (indicating “NOK” events). These results align with the box plot of consecutive day numbers for the “OK/NOK” event. Therefore, we can draw a similar conclusion as mentioned earlier for the Magallanes region: the early and robust implementation of phase 1 affects the duration of the “OK” event, indicating that the median time spent in the “OK” event is greater than in the “NOK” event.

In an attempt to clarify these results, an observation stems from the graphs depicting the percentage of individuals in each region during phases 1 and 2. It becomes apparent that, in the Metropolitan region, the initiation of protocols occurred after the triggering of *R_r,t_*. This observation remains consistent, even though these curves are influenced by or closely track the pattern of the curve representing the percentage of confirmed cases (blue curve), which is unlike the situation in the Magallanes region. Further analysis is warranted to investigate the mid-June period in more detail.

## Conclusion

In this article, we conduct an in-depth examination of the impact of COVID-19 quarantines initiated by the Chilean Ministry of Health since the country’s first confirmed positive case. Through a comprehensive analysis, employing both descriptive and residual lifetime methods, and utilizing data from authoritative sources accessible at https://www.gob.cl/pasoapaso/cifrasoficiales/ (accessed on April 22, 2021), we draw significant insights regarding the efficacy of these measures.

In our initial analysis, we examined the time frames denoted by the implementation of phase one and phase two quarantines, which are represented by the black and brown curves in Fig 1. It is apparent that these measures do not immediately result in a reduction of COVID-19 cases, as shown by the blue curve.

Then we proceeded to compare the means, it was proven that there are no statistically significant differences (p-value > 0.05, as determined by classical t-tests and Wilcoxon tests) between the number of consecutive days where *R_r,t_* ≤ 1 and *R_r,t_* > 1 across different regions (see Fig 2).

Looking at the data descriptively, when examining the distribution of residual times for events *R_r,t_* ≤ 1 and *R_r,t_* > 1, we observed that the effects of quarantine measures varied significantly across different regions in Chile, as depicted in Fig 1. Notably, the residual times for the Antofagasta, Atacama, Metropolitan, and Biobio regions stood out as significantly different, indicating that the mitigation measures in these regions were less effective compared to other regions. These findings strongly indicate that the effective control of the virus’s spread was not achieved. This conclusion is consistent with similar results reported by Jerez-Lillo et al. (2022) [9], which highlight the uncertain effectiveness of quarantine measures due to the absence of a robust epidemiological foundation. Additionally, Barría-Sandoval et al. (2022) [8] concluded that, in certain regions, the decision-making process regarding quarantine measures did not align with infection rates, resulting in limited reductions in infections. Grebe et al. (2020) [11] also noted that quarantine durations in Chile were primarily determined by the Ministry of Health without consideration of specific parameters, relying on the number of active cases per municipality. In contrast, Kristjanpoller et al. (2021) [10] suggested that dynamic quarantine measures in Chile initially helped reduce contagion rates but became less effectivene over longer periods.

Utilizing residual time with respect to the *R_r,t_* index state provides us with a means to estimate the duration of the *R_r,t_* > 1 and *R_r,t_* ≤ 1 conditions after a specified time t0 has elapsed. If the duration of *R_r,t_* > 1 exceeds that of *R_r,t_* ≤ 1, it strongly suggests that the measures implemented to attain the desired outcome of reversing the prior condition have not been effective.

In Fig 3, the panel labels representing the names different regions are color-coded as green, yellow, and red, indicating the impact of the measures for controlling the pandemic on the mean and median residual time. In this context, the green color signifies that, in general, the curve for mean or median residual time over *R_r,t_* > 1 (orange/cyan color) is less than the corresponding curve over *R_r,t_* ≤ 1 (green/violet color). The yellow where we have not observed a significant increase in the mean or median residual time curve over *R_r,t_* > 1 compared to *R_r,t_* ≤ 1. Conversely, the red color suggests that, on the whole, the mean or median residual time curve over *R_r,t_* > 1 is biger than that over *R_r,t_* ≤ 1.

Building upon above analysis and considering the information presented in Fig 1, a noticeable pattern emerges. In regions where the proportion of confirmed COVID-19 cases remains nearly negligible during an initial period exceeding one month, and subsequently by experiencing a gradual increase, the mean or median residual time can be classifed using yellow or green coloring. This observation generally holds true for most regions, although there is an exception in the case of the Ñuble region.
